# Synergistic Potential of Antimicrobial Combinations Against Methicillin-Resistant *Staphylococcus aureus*

**DOI:** 10.3389/fmicb.2020.01919

**Published:** 2020-08-17

**Authors:** Yang Yu, Han-Liang Huang, Xin-Qing Ye, Da-Tong Cai, Jin-Tao Fang, Jian Sun, Xiao-Ping Liao, Ya-Hong Liu

**Affiliations:** ^1^Guangdong Provincial Key Laboratory of Veterinary Pharmaceutics Development and Safety Evaluation, South China Agricultural University, Guangzhou, China; ^2^National Risk Assessment Laboratory for Antimicrobial Resistance of Animal Original Bacteria, South China Agricultural University, Guangzhou, China

**Keywords:** synergism, MRSA, vancomycin, combination therapy, *in vivo* model

## Abstract

The chemotherapeutic options for methicillin-resistant *Staphylococcus aureus* (MRSA) infections are limited. Due to the multiple resistant MRSA, therapeutic failure has occurred frequently, even using antibiotics belonging to different categories in clinical scenarios, very recently. This study aimed to investigate the interactions between 11 antibiotics representing different mechanisms of action against MRSA strains and provide therapeutic strategies for clinical infections. Susceptibilities for MRSA strains were determined by broth microdilution or agar dilution according to CLSI guideline. By grouping with each other, a total of 55 combinations were evaluated. The potential synergism was detected through drug interaction assays and further investigated for time-killing curves and an *in vivo* neutropenic mouse infection model. A total of six combinations (vancomycin with rifampicin, vancomycin with oxacillin, levofloxacin with oxacillin, gentamycin with oxacillin, clindamycin with oxacillin, and clindamycin with levofloxacin) showed synergistic activity against the MRSA ATCC 43300 strain. However, antibacterial activity against clinical isolate #161402 was only observed when vancomycin combined with oxacillin or rifampicin in time-killing assays. Next, therapeutic effectiveness of vancomycin/oxacillin and vancomycin/rifampicin was verified by an *in vivo* mouse infection model inoculated with #161402. Further investigations on antimicrobial synergism of vancomycin plus oxacillin and vancomycin plus rifampicin against 113 wild-type MRSA strains were evidenced by combined antibiotic MICs and bacterial growth inhibition and *in vitro* dynamic killing profiles. In summary, vancomycin/rifampicin and vancomycin/oxacillin are the most potential combinations for clinical MRSA infection upon both *in vitro* and *in vivo* tests. Other synergetic combinations of levofloxacin/oxacillin, gentamycin/oxacillin, clindamycin/oxacillin, and clindamycin/fosfomycin are also selected but may need more assessment for further application.

## Introduction

The inappropriate use and overuse of antibiotics have facilitated the emergence of drug-resistant or even multiple-drug-resistant (MDR) *Staphylococcus aureus* worldwide (Rodríguez-Lázaro et al., 2017). Methicillin-resistant *S. aureus* (MRSA) is a common pathogen for nosocomial infections and exhibits essential resistance to methicillin, oxacillin, nafcillin, carbapenems, and other β-lactams. For now, the clinical therapies against MRSA infection are limited to a few antimicrobial agents, such as ceftaroline, new cephalosporins, retaining significant activity against *S. aureus* and even MRSA strains; and linezolid belonging to the oxazolidinone class and approved for *S. aureus* infections in clinics (Saxena et al., 2019). However, due to the rapid evaluation of antimicrobial resistance, MRSA strains have possessed reduced susceptibilities to vancomycin, daptomycin, levofloxacin, clindamycin, and sulfamethoxazole (Richter et al., 2011). Even worse, the simultaneous resistance to vancomycin, daptomycin, and ceftaroline has been identified in MRSA recently (Wüthrich et al., 2019). Given that the monotherapy is limited in clinical treatment and the new drug development is a lengthy process, the combination therapy has currently become one of the most effective approaches against bacterial infections benefiting from the enlarged spectrum, enhanced antibacterial activity, minimized doses, and reduced drug toxicity of antibiotic combinations. For instance, combination treatments of vancomycin or tigecycline with rifampicin are successful in treatment of many cases (Vergidis et al., 2015). Fosfomycin is a promising option to treat infections caused by multi-drug resistant (MDR) pathogens when combining with daptomycin or β-lactams (Coronado-Álvarez et al., 2019).

In the current study, a total of 11 antibiotics with different mechanisms of antibacterial activity (inhibiting the synthesis of cell wall, protein or DNA, respectively) were selected and combined with each other to examine the pairwise interactions and identify the synergistic combinations against MRSA strains. After the preliminary screening, combinations of oxacillin with levofloxacin, oxacillin with vancomycin, oxacillin with gentamycin, oxacillin with clindamycin, and vancomycin with rifampicin exhibited the collateral effect on the MRSA ATCC 43300 strain. The further experimental verification elucidated that vancomycin combined with oxacillin or rifampicin has synergistic antibacterial activity against the clinical wild-type MRSA strain both *in vitro* and *in vivo*.

## Materials and Methods

### Reagents and Bacterial Strains

Antibiotics of levofloxacin, tigecycline, vancomycin, fosfomycin, linezolid, oxacillin, rifampicin, clindamycin, gentamycin, daptomycin, and chloramphenicol were selected as the representative agents from different categories of antimicrobial agents (Supplementary Table S1). The antimicrobial susceptibility testing (AST) of 11 antibiotics was performed according to the CLSI guideline for the 113 clinical MRSA strains isolated from hospitals in Guangzhou, China (CLSI, 2018). The *S. aureus* ATCC 29213 was used for the quality control and the MRSA ATCC 43300 was used as the standard strains. The MRSA clinical strain #161402, with multiple resistance to tigecycline, fosfomycin, levofloxacin, oxacillin, rifampicin, clindamycin, and gentamycin, was used in the *in vitro* and *in vivo* experiments to test the therapeutic effectiveness of drug combinations. The Mueller Hinton (MH) broth and agar were used for AST and the Lysogeny broth (LB) and agar were used for drug interaction assays. The Mannitol salt agar (MSA) was used to identify *S. aureus* strains by the gold and yellow color of bacterial colony.

### Determination of Single-Drug Concentration

Single-drug concentrations were determined as doses inhibiting bacterial growth. The mid-log cultures of the MRSA ATCC 43300 strain were diluted to 5 × 10^5^ cfu/mL and exposed to LB broth with gradient-diluted antibiotics. The mixtures were incubated overnight at 37°C. After incubation, 200 μL of culture samples were added to 96-well cell incubating plates, and the OD_600_ values were determined using an Ensight^TM^ Multimode Plate Reader (PerkinElmer, Waltham, MA, United States). The OD_600_ value of bacterial growth in drug-free medium was used as the normalization standard. The drug concentrations that were able to inhibit 10–50% of bacterial growth were considered as the potential single-drug concentrations and were used in the following experiments.

### Drug Interaction Assays

The interactions of combined antibiotics were investigated as the previous description with minor modification (Yeh et al., 2006). In brief, tubes containing 8 mL of LB broth of mid-log bacterial cultures were mixed with the following four administration options for each combination: (i) 2 mL fresh LB broth as growth control; (ii) 2 mL stock of drug X to measure the growth rate of X singly; (iii) 2 mL stock of drug Y to measure the growth rate of Y singly; and (iv) 1 mL stock of drug X and 1 mL stock of drug Y to measure the combined growth rate. After the overnight incubation, OD_600_ values of all the incubations were determined as described above. The OD_*x*_, OD_*y*_, OD_*xy*_, and OD_*control*_ are representing the groups of drug X and Y singly, the combination of X and Y, and the growth control in the absence of any drugs. The OD_*control*_ was used as the standard normalization for measuring the growth rates of administration groups, where

$$W_{x}=\frac{O\,D_{x}}{O\,D_{c\,o\,n\,t\,r\,o\,l}},W_{y}=\frac{O\,D_{y}}{O\,D_{c\,o\,n\,t\,r\,o\,l}}\,W_{x\,y}=\frac{O\,D_{x\,y}}{O\,D_{c\,o\,n\,t\,r\,o\,l}}.$$

The index of drug interaction ($\tilde{\varepsilon }$) was classified using the following equations as previously described (Yeh et al., 2006):

$$\tilde{\varepsilon }=\frac{\left(W_{x\,y}-W_{x}\,W_{y}\right)}{\left|\tilde{W_{x\,y}}-W_{x}\,W_{y}\right|},$$

where $\tilde{W_{x\,y}}=\min\left[W_{x},W_{y}\right]$ if *W*_*xy*_ > *W*_*x*_*W*_*y*_, or $\tilde{W_{x\,y}}=0$ if *W*_*xy*_≤*W*_*x*_*W*_*y*_;

$$\tilde{\varepsilon }=\frac{\left(W_{x\,y}-\min\left[W_{x},W_{y}\right]\right)}{\left(1-m\,i\,n\,\left[W_{x}-W_{y}\right]\right)}+1,$$

where *W*_*xy*_ > *min*⁡[*W*_*x*_,*W*_*y*_].

For $\tilde{\varepsilon }$ < -0.5, the interaction is considered as synergistic; $\tilde{\varepsilon }$ > 0.5 as antagonistic; otherwise the interaction is scored as additive. A mid-log bacterial density of MRSA ATCC 43300 was used in this experiment, and the concentrations of antibiotics were recommended as above.

### *In vitro* Time-Killing Curves

Illustrated by drug interaction assays, the synergistic combinations (vancomycin/oxacillin, vancomycin/rifampicin, levofloxacin/oxacillin, gentamycin/oxacillin, clindamycin/oxacillin, and clindamycin/fosfomycin) were tested for *in vitro* killing activity against ATCC 43300 and the MRSA clinical isolate #161402. The mid-log cultures of *S. aureus* strains were appropriately diluted to achieve an initial cell density of 10^6^ cfu/mL and then exposed to the drug-free, single drug X/Y, and combination of X and Y medium, respectively. The colony counts were then detected and calculated at 3, 6, 9, 24, 27, 48, and 72 h. The concentrations of vancomycin, oxacillin, rifampicin, levofloxacin, gentamycin, clindamycin, and fosfomycin were 2, 1 or 10, 0.03, 0.25, 512, 512, and 320 mg/L respectively, according to the MICs distribution for MRSA strains (Supplementary Figure S2).

### *In vivo* Synergism

The neutropenic mouse thigh model was employed for testing the *in vivo* synergistic efficacy of the following drug combinations: vancomycin plus rifampicin and vancomycin plus oxacillin, referring to the considerable synergism against both wild-type and standard MRSA strains upon time-killing curves. The 6-week-old SPF female ICR mice weighing 25 ± 2 g were administered with cyclophosphamide (Yuanye Biotechnology, Shanghai, China) to induce neutropenia (neutrophils ≤ 100/mm^3^) as previously described (Yu et al., 2019). Briefly, an initial dose of 150 mg/kg of cyclophosphamide was injected intraperitoneally daily for 4 days and followed by a single dose of 100 mg/kg on the fifth day. The mid-log bacterial cultures were appropriately diluted by normal saline, and the neutropenic mice were then intramuscularly injected 100 μL of bacterial suspension (10^7^ cfu/mL) into each posterior thigh muscle. After a 1 h, a placebo (normal saline, Group I) or antibiotics was administered in the following manner: single-drug groups received only Drug A (vancomycin, Group II) or Drug B (rifampicin or oxacillin, Group III), and combined groups received both A and B (vancomycin in combination with rifampicin or oxacillin, Group IV). Dosing regimens were 2 mg/kg for vancomycin administrated intraperitoneally, 0.03 mg/kg rifampicin intragastrically, and 1 mg/kg oxacillin subcutaneously, and the injection volume was 100 μL for all drugs. After 24 h, groups of mice were sacrificed, and thigh homogenates in sterile normal saline were sampled for bacterial burden quantifications. In each group, three or four mice were used, and a total of 6 or 8 thigh samples from each group were collected. Both MRSA ATCC 43300 and #161402 strains were tested in this experiment. The significant differences between groups were analyzed using one-way ANOVA, followed by Dunnett’s multiple comparisons test using GraphPad Prism 7.0 (La Jolla CA, United States).

### Ethics Statement

The SPF female ICR mice were purchased from Hunan Silaikejingda Lab Animal (Hunan, China). Breeding was conducted under SPF conditions. The mice were housed at four per cage with 12-h light:dark cycles and fed SPF food and water *ad libitum*. The *in vivo* mouse study was approved by the Animal Care and Use Committee of South China Agricultural University and followed the Guangdong Laboratory Animal Welfare and Ethics guidelines [GB 14925-2010, SYXK (Guangdong) 2014-0316].

### Verification of Combined Antibacterial Effect

To further claim the therapeutic effectiveness of combined antibiotics, sub-inhibitory concentrations of vancomycin (0.5 mg/L) and oxacillin (1 mg/L) and rifampicin (0.03 mg/L) were applied in a series of *in vitro* antibacterial tests against the total 113 wild-type MRSA strains. Firstly, the MICs of vancomycin (in the presence of 1 mg/L oxacillin or 0.03 mg/L rifampicin) and oxacillin (in presence of 0.5 mg/L vancomycin) and rifampicin (in presence of 0.5 vancomycin) were evaluated by agar dilution and compared with single-drug MICs. Secondly, bacterial growth rates in groups of drug-free and monotherapy (oxacillin or rifampicin or vancomycin) and combined therapy (vancomycin/oxacillin or vancomycin/rifampicin) were estimated and calculated. Thirdly, dynamic characteristics of antimicrobial activity were estimated by time-killing curves for 24 h. Details of the procedures were described above. Statistical analysis was assessed using biological replicates (*n* = 113).

## Results

### The MICs and Single Drug Concentrations

In Table 1, The examined 113 clinical strains were highly resistant to levofloxacin showing MIC_50_ and MIC_90_ of 4 and 128 mg/L, oxacillin of 4 and 64 mg/L, clindamycin of ≥256 and ≥256 mg/L, gentamycin of 64 and ≥256 mg/L, and chloramphenicol of 64 and 128 mg/L. MIC distribution of rifampicin showed two sub-populations with MIC <0.125 mg/L and 0.25 ≤ MIC ≤ 256 mg/L, respectively. Antibiotics of tigecycline, vancomycin, linezolid, and daptomycin were susceptible against the most MRSA isolates. MDR strains with resistance to levofloxacin, linezolid, oxacillin, rifampicin, clindamycin, gentamycin, and chloramphenicol were detected in this study as well.

**TABLE 1:** MIC distributions for MRSA strains used in this study.

Antimicrobial agents	MIC distribution by the number of isolates (mg/L)																	MIC_50_	MIC_90_	Resistant breakpoints*
	≤0.004	0.008	0.015	0.03	0.1	0.13	0.3	0.5	1	2	4	8	16	32	64	128	≥256			
Levofloxacin	0	0	0	0	0	0	4	**2**	0	**37**	**35**	**10**	**3**	**9**	**1**	**4**	**8**	4	128	≥4
Tigecycline	0	0	0	0	0	0	**4**	**9**	**9**	**63**	**20**	**2**	**4**	**1**	**1**	0	0	2	4	NA
Vancomycin	0	0	0	0	0	0	0	0	**61**	**45**	**7**	0	0	0	0	0	0	1	2	≥16
Fosfomycin	0	0	0	0	0	0	0	0	0	**2**	**6**	**19**	**38**	**22**	**5**	**2**	**19**	16	≥256	NA
Linezolid	0	0	0	0	0	0	**8**	**21**	**64**	**17**	0	0	0	0	**1**	**0**	**2**	1	2	≥8
Oxacillin	0	0	0	0	0	0	**6**	**7**	**14**	**23**	**20**	**22**	**5**	**2**	**4**	**6**	**4**	4	64	≥0.5
Rifampin	**1**	**13**	**15**	**29**	**3**	0	**1**	**1**	**4**	**5**	**8**	**2**	**2**	**3**	**1**	**12**	**13**	0.03	≥256	≥4
Clindamycin	0	0	0	0	0	0	0	0	0	0	0	0	0	0	0	**2**	**111**	≥256	≥256	≥4
Gentamycin	0	0	0	0	0	**1**	0	**8**	**3**	**1**	**1**	**1**	**6**	**29**	**26**	**20**	**17**	64	≥256	≥16
Daptomycin	0	0	0	0	**29**	**55**	**8**	**13**	0	0	0	**1**	0	0	0	0	**7**	0.12	0.5	≤1^*a*^
Chloramphenicol	0	0	0	0	0	0	0	0	0	**1**	**2**	**1**	**9**	**8**	**68**	**16**	**8**	64	128	≥32

*Resistant breakpoints refer to CLSI documents (M100 2019). ^*a*^Susceptible with MIC ≦ 1 mg/L. NA, not applicable; Red vertical line for breakpoints. Bold values represent the number of strains.

Single drug concentrations that caused 10–50% inhibition of bacterial growth of *S. aureus* ATCC 43300 were evaluated and shown in Supplementary Table S1 and Supplementary Figure S1. For rifampicin, clindamycin, and gentamycin, the maximum of 20% inhibition was observed when given 0.5- to 1-fold MICs. Subinhibitory concentrations of most antibiotics only achieved 30–40% growth reduction, like oxacillin, linezolid, levofloxacin, daptomycin, and chloramphenicol. Notably, the bacteriostatic activity of drugs did not progressively increase with dosing concentrations, which might be due to the characteristics of antibiotics. Sub-MICs of antibiotics used in the drug interaction assays were shown in Supplementary Table S2.

### Evidence of Synergism

**FIGURE 1:**
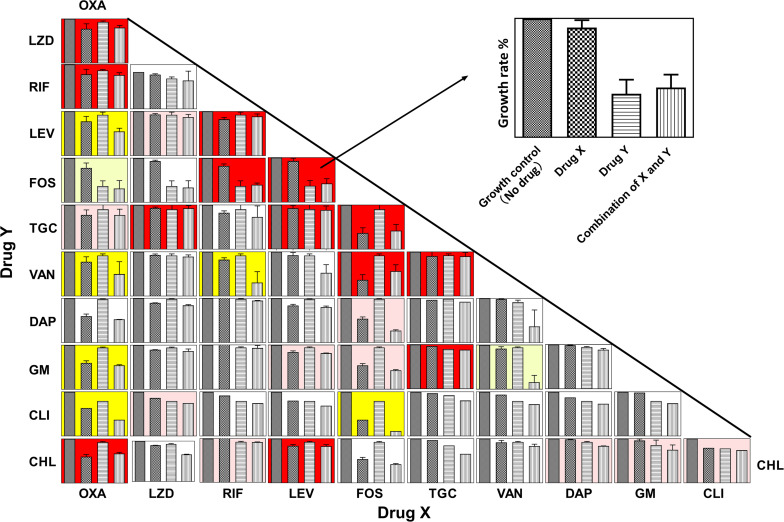
Panels of pairwise combinations for 11 antibiotics. Growth rates of no-drug, drug X only, drug Y only, and combinations of X and Y are shown in each panel (Upper right corner: zoom in on each panel). Error bars represent variability in replicate measurements. Synergism ($\tilde{\varepsilon }$ < −0.5), antagonistic ($\tilde{\varepsilon }$ > 0.5), and additive (−0.25 < $\tilde{\varepsilon }$ < 0.5 and −0.5 < $\tilde{\varepsilon }$ < 0.25) effects are labeled with yellow, red, and white backgrounds in the corresponding panels. Panels with pink and light-yellow color represent antagonistic buffering (0.25 < $\tilde{\varepsilon }$ < 0.5) and mild synergistic interactions (−0.5 < $\tilde{\varepsilon }$ < −0.25), respectively.

Figure 1 shows the panels of drug interactions which were arrayed in a matrix and painted with colors representing synergistic, antagonistic, or additive effect. Among the total 55 pairwise interactions, 6 combinations exhibited synergistic efficacy against ATCC 43300 ($\tilde{\varepsilon }$ < -0.5) and 13 pairwise interactions showed an antagonistic effect ($\tilde{\varepsilon }$ > 0.5). Oxacillin exhibited a potential synergism in combination with levofloxacin, vancomycin, gentamycin, and clindamycin. Interestingly, antagonistic buffering and synergistic buffering were also observed in combined interactions indicated as pink and light yellow panels. The additive or indifferent effects were shown by most of the antibiotic combinations as illustrated in white background.

### *In vitro* Effects of Antibiotic Challenge

To test the bacterial responses to drug combinations, killing curves of six pairwise synergistic combinations were evaluated against both the ATCC 43300 strain and clinical MRSA isolate #161402. During the 48 h of exposure, single-drug groups barely exerted the killing activity against either ATCC 43300 or #161402. When administrated with combined drugs, bacterial count reduction of 2–3 log cfu/mL was observed for six pairwise regimens against ATCC 43300. However, combinations of levofloxacin plus oxacillin, gentamycin plus oxacillin, clindamycin plus oxacillin, and clindamycin plus fosfomycin showed insufficient killing activity against isolate #162402. In contrast, the combinations of vancomycin plus oxacillin or rifampin showed considerable inhibition against #161402 at the first 24 h, but regrowth was observed in the following 48 h (Supplementary Figure S2). In consideration of the MIC distributions and the *in vitro* killing activity, combinations of vancomycin with oxacillin or rifampicin were selected for the further antibacterial evaluation.

### *In vivo* Synergistic Efficacy

**FIGURE 2:**
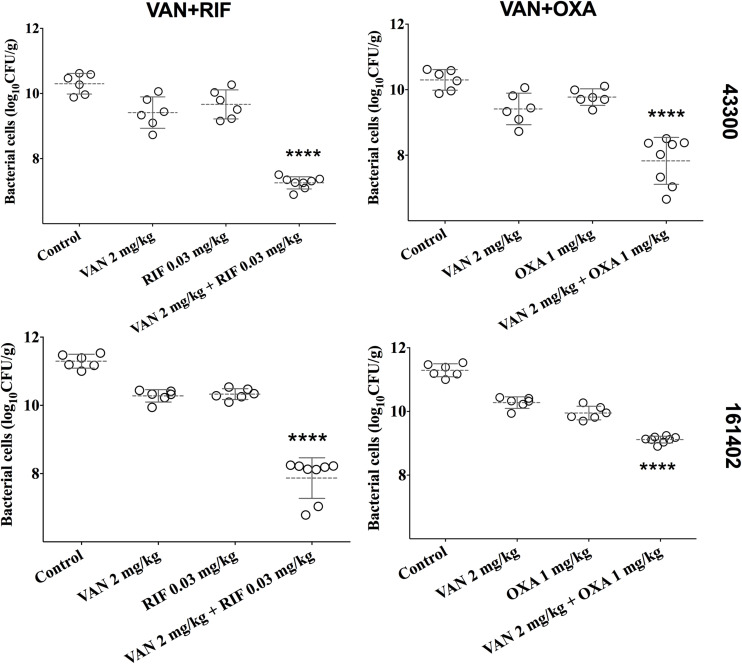
Bacterial densities from mouse thigh muscles (log cfu/g) after 24 h of monotherapy or combination therapy for the two pairwise drugs against ATCC 43300 and #161402, respectively. ****P < 0.001 by the one-way ANOVA.

We developed a murine infection model and used ATCC 43300 and #161402 strains to further evaluate the *in vivo* antibacterial efficacy of vancomycin in combination with oxacillin or rifampicin. The bacterial growth in the control groups increased to over 10-log cfu/g at 24 h after inoculation (Figure 2) but was inhibited to 7–8 log cfu/g when applying a combination therapy of vancomycin plus rifampicin against both ATCC 43300 and #161402. In addition, the combination of vancomycin plus oxacillin showed a significant decrease in bacterial growth compared with the control groups (*P* < 0.001). Monotherapy of vancomycin, rifampicin, and oxacillin barely inhibited the growth of these two strains, although lower bacterial counts were observed in groups of vancomycin injected alone. Significantly enhanced activity given synergistic combinations was elucidated when compared with the other groups of monotherapy (*P* < 0.05).

### Evidence of Synergistic Effect

**FIGURE 3:**
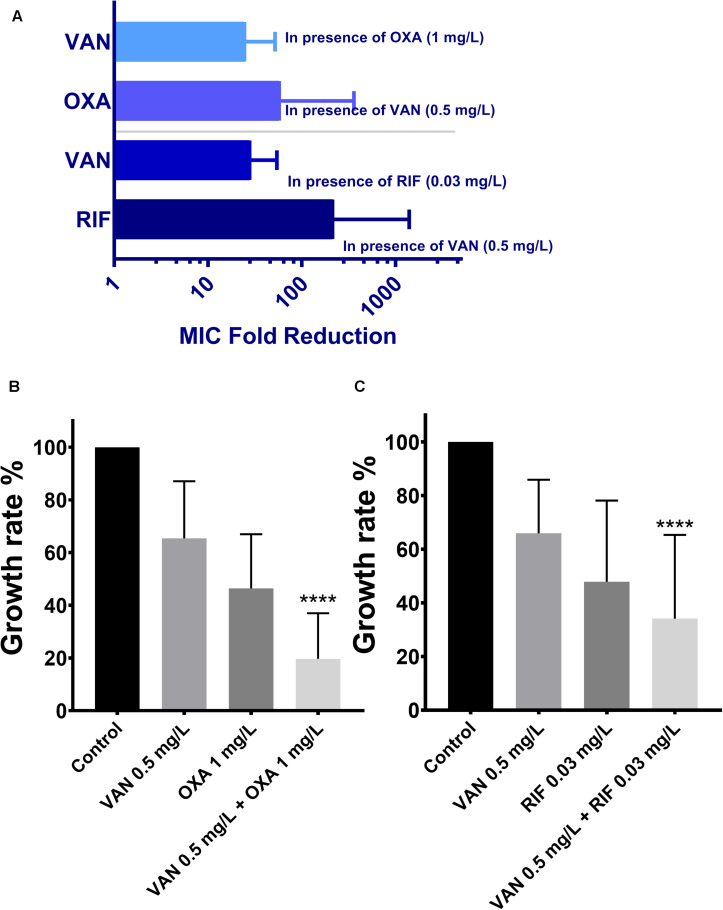
The therapeutic effectiveness of vancomycin combining oxacillin and vancomycin combining rifampicin against the 113 wild-type MRSA strains. The error bars were estimated by 113 biological replicates. **(A)** Fold reduction of MICs in presence of the partnering antibiotics. **(B,C)** Growth rates of drug-free, vancomycin only, oxacillin only, rifampicin only, combination of vancomycin and oxacillin, and combination of vancomycin and rifampicin. ****Significant difference between combination groups and other groups (control and single drug); *P* < 0.0001, one-way ANOVA.

**FIGURE 4:**
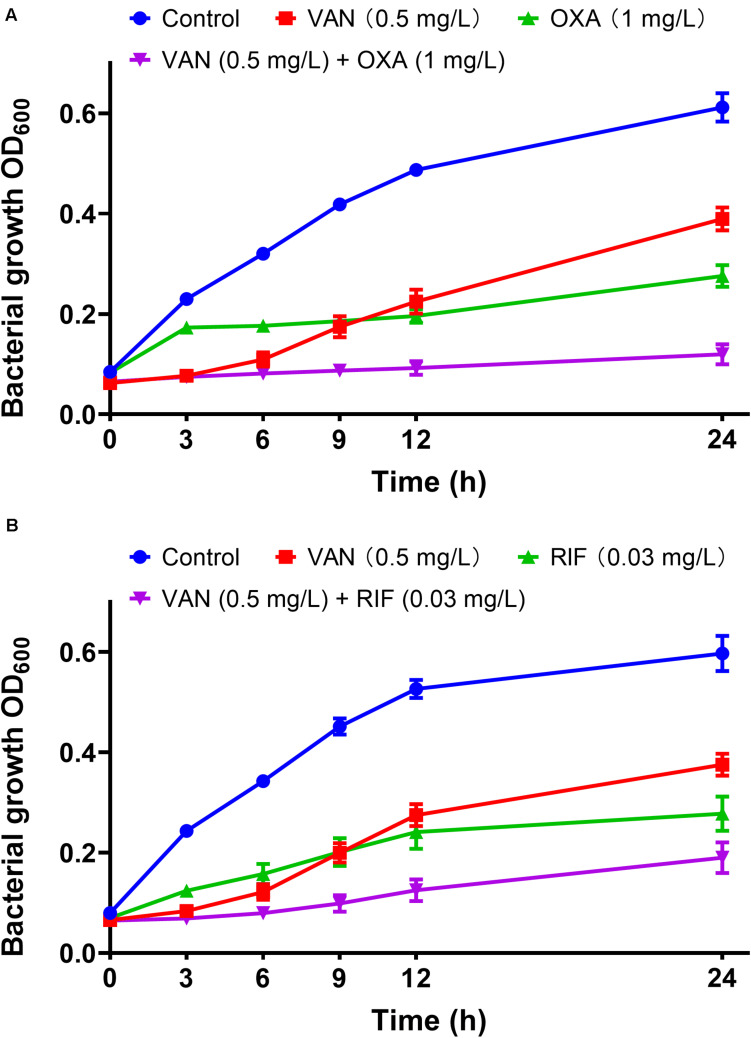
*In vitro* dynamic killing tests of combinations of vancomycin plus oxacillin **(A)** and vancomycin and rifampicin **(B)** against the 113 wild-type MRSA strains. Sub-inhibitory concentrations of 0.5 mg/L (vancomycin), 1 mg/L (oxacillin), and 0.03 mg/L (rifampicin) were used. The error bar was calculated based on the biological repetitions (*n* = 113).

MICs of vancomycin and oxacillin and rifampicin against 113 wild-type MRSA strains were re-estimated by agar dilution in the presence of the corresponding partnering antibiotics. The magnitude of MIC reduction for each antibiotic when used alone vs. in combination with partners was expressed as a fold change (Figure 3A). Notably, when combined with 1 mg/L oxacillin or 0.03 mg/L rifampicin, the MIC of vancomycin dropped nearly 50-fold. On the other hand, the fold-reductions in MICs of oxacillin and rifampicin were >160 and >100, respectively, with an addition of 0.5 mg/L vancomycin. Antimicrobial activity of combinations of vancomycin plus oxacillin and vancomycin plus rifampicin was confirmed by inhibition of bacterial growth (Figures 3B,C). When vancomycin combined with oxacillin or rifampicin, the bacterial growth rates were <20% or <40%, which are significantly lower than those of the groups that used single drugs (*P* < 0.0001, one-way ANOVA). In addition, *in vitro* killing curves (Figure 4) explained the dynamic antibacterial activity of antibiotic combinations. During 24-h incubation, MRSA strains were inhibited by vancomycin combined with oxacillin or rifampicin, but a slight regrowth was detected using vancomycin plus rifampicin.

## Discussion

Numerous experimental and clinical studies have demonstrated that MRSA strains show basal resistance to methicillin, oxacillin, nafcillin, carbapenems, and other β-lactams (Islam et al., 2019). Currently, MRSA strains are mostly susceptible to vancomycin, daptomycin, and linezolid, the preferred antimicrobial agents for clinical therapies. Primarily by inhibiting the cell wall synthesis, vancomycin shows therapeutic activity against MRSA (Howden et al., 2010). Daptomycin is a cyclic lipopeptide and represents a second generation of glycopeptide antibiotics that are effective against MRSA (Sader et al., 2014). Linezolid has become an important antimicrobial against gram-positive bacteria including MRSA and inhibits the translation by binding to the 23S rRNA peptidyl transferase region (Hashemian et al., 2018). In our study, the MIC distribution indicated that most clinical isolates were susceptible to vancomycin, daptomycin, and linezolid, which was consistent with the previous study (Kates et al., 2018). Similar to the investigation in other countries, we also found high-level resistance to oxacillin and gentamycin in our tested bacterial population, but MICs of clindamycin and chloramphenicol were different (Sohail and Latif, 2018; Syed et al., 2019). For instance, MRSA isolates from Malaysia, resistant to β-lactams mediated by the PBP_2__*a*_ encoding *mecA* gene, showed high resistance to gentamycin but less to clindamycin (only 31.3%) and moderate resistance to chloramphenicol (Hamzah et al., 2019). On the contrary, the clinical MRSA isolates collected from Guangzhou were highly resistant to clindamycin and chloramphenicol, suggesting a more developed situation of antimicrobial resistance.

By screening the conventional antibiotics used for MRSA infection, we found that vancomycin/oxacillin and vancomycin/rifampicin displayed synergistic effects on MDR-MRSA isolates by both the *in vitro* killing trials and the *in vivo* mouse model. The synergism of vancomycin and the β-lactams has been reported previously as well and achieved significantly lower rates of treatment failure than monotherapy of vancomycin against MRSA (Truong et al., 2018). Another study demonstrated that the combination of vancomycin and oxacillin showed synergism against three methicillin-resistant vancomycin-intermediate *S. aureus* (VISA) strains and one heterogeneous VISA (hVISA) strain (Pharmaceuticals et al., 2013). The potential synergism may be that vancomycin can easily get into the bacterial cell with the assistance of β-lactams by providing a pathway for entry (Lewis et al., 2018), and the combination of vancomycin and β-lactams down-regulates the expression levels of *mecA* gene in MRSA isolates (Abdolahi and Khodavandi, 2019). In addition, recently, a new theory of collateral susceptibility in antimicrobial agents may inspire a novel insight for the synergism of vancomycin combined with oxacillin. Previous studies reported the oxacillin MICs of *S. aureus* strains decreased after vancomycin treatment (Wang et al., 2017), so called “see-saw phenomenon” occurring in certain stages of vancomycin resistance promotion, suggesting that upon acquisition of vancomycin resistance or VISA evolution, some strains show a concomitant decrease in oxacillin resistance (Bhateja et al., 2006). It was reported that mutated *graR* may impair oxacillin resistance (Neoh et al., 2008). The synergistic effect of vancomycin/rifampicin was not only reported in this study, but also in treatment of the non-nosocomial healthcare-associated infective endocarditis (NNHCA-IE) caused by MRSA strain USA 400/SCC mec IV (Damasco et al., 2013). Given that rifampicin interferes with the DNA synthesis while vancomycin disrupts the bacterial cell wall synthesis, the drug combination may disturb cell reproduction in different stages. For example, when vancomycin combined with rifampicin, significantly higher cell damage and decrease in biofilms thicknesses were detected (Boudjemaa et al., 2017).

As shown in Figure 1 and Supplementary Figure S2, the synergistic activities of levofloxacin/oxacillin, gentamycin/oxacillin, clindamycin/oxacillin, and chloramphenicol/fos fomycin were limited. In the *in vitro* killing curves, these combinations showed no antibacterial activity against clinical isolate #162402 which was highly resistant to levofloxacin, gentamycin, clindamycin, and fosfomycin with MICs over 256 mg/L. The phenomenon indicated that high resistance to the component of pairwise antibiotics would affect the combined effect, and the ideal situation is that the target pathogens are not highly resistant to either component of the combinations.

In this study, we also found 13 pairwise combinations that showed antagonistic effects ($\tilde{\varepsilon }$_*min*_ > 0.5) and 10 pairwise interactions that exhibited lower antagonistic effects (Figure 1, red and pink panels). However, some of these combinations showed synergism against MRSA strains in other studies. For example, the combination of daptomycin plus fosfomycin were synergistic in the treatment of experimental endocarditis caused by MRSA strains by both *in vitro* and *in vivo* studies (Garcia-de-la-Maria et al., 2018), and combinations of fosfomycin and rifampin (or tigecycline) have synergistic antibacterial activity in a mouse wound infection model (Simonetti et al., 2018). However, individual differences including serotype, virulence, and antimicrobial resistance must be considered in the evaluation of antibacterial activity, especially for *in vivo* treatment.

## Conclusion

In conclusion, vancomycin combined with oxacillin or rifampicin was detected as synergistically effective against MRSA infections among a matrix screening of antibiotic combinations. The efficacy of these two combinations was further confirmed by an *in vivo* neutropenic mouse thigh model against a clinical MRSA isolate, suggesting that vancomycin/oxacillin and vancomycin/rifampicin are potential strategies for the treatment of MRSA infections. Studies focusing on the synergism and the mechanism of the combinations should be further investigated for understanding the drug interactions.

## Data Availability Statement

All datasets generated for this study are included in the article/Supplementary Material.

## Ethics Statement

The animal study was reviewed and approved by the Animal Care and Use Committee of the South China Agricultural University.

## Author Contributions

Y-HL and YY conceived of the study and designed the experiment. X-QY and YY drafted the manuscript. H-LH and J-TF carried out the *in vitro* experiments and analyzed relative data. JS, D-TC, and X-PL revised the manuscript. All authors contributed to the article and approved the submitted version.

## Conflict of Interest

The authors declare that the research was conducted in the absence of any commercial or financial relationships that could be construed as a potential conflict of interest.
