# Supplementary material for: Synergistic Potential of Antimicrobial Combinations Against Methicillin-Resistant Staphylococcus aureus
Source: Front Microbiol. 2020 Aug 17;11:1919. doi: 10.3389/fmicb.2020.01919 (PMC7461988; doi:10.3389/fmicb.2020.01919)
Supplement: Supplementary file 1 [file Data_Sheet_1.docx]

**Appendix.**

**Table S1.** The categories, abbreviation, and mechanism of action of the antimicrobial agents used in this study.

| **Drug** | **Categories** | **Abbr.** | **Main mechanism(s) of action** |
| --- | --- | --- | --- |
| Oxacillin | Penicillins | OXA | Cell wall synthesis |
| Vancomycin | Glycopeptides | VAN | Cell wall synthesis |
| Fosfomycin | Fosfomycines | FOS | Cell wall synthesis |
| Levofloxacin | Fluoroquinolones | LEV | DNA helicase |
| Daptomycin | Lipopeptide | DAP | Cell wall peptide |
| Tigecycline | Tetracyclines | TGC | Protein synthesis, 30S |
| Gentamycin | Aminoglycosides | GM | Protein synthesis, 30S |
| Rifampicin | Rifomycins | RIF | DNA transcription synthesis RNA |
| Linezolid | Oxazolidinones | LZD | Protein synthesis, 50S |
| Clindamycin | Lincosamides | CLI | Protein synthesis, 50S |
| Chloramphenicol | Amphhenicols | CHL | Protein synthesis, 50S |

**Table S2.** Reduction of bacterial growth rate versus different drug concentrations.

Drug concentrations were shown as fold-MICs. OXA, oxacillin; LZD, linezolid; RIF, rifampicin; FOS, fosfomycin; TEG, tigecycline; VAN, vancomycin; DAP, daptomycin, GM, gentamycin; CLD, clindamycin; CHL, chloramphenicol. —, cannot achieve the corresponding rates with the tested drug concentrations; bold, drug concentrations (fold-MICs) tested in drug interaction assays.


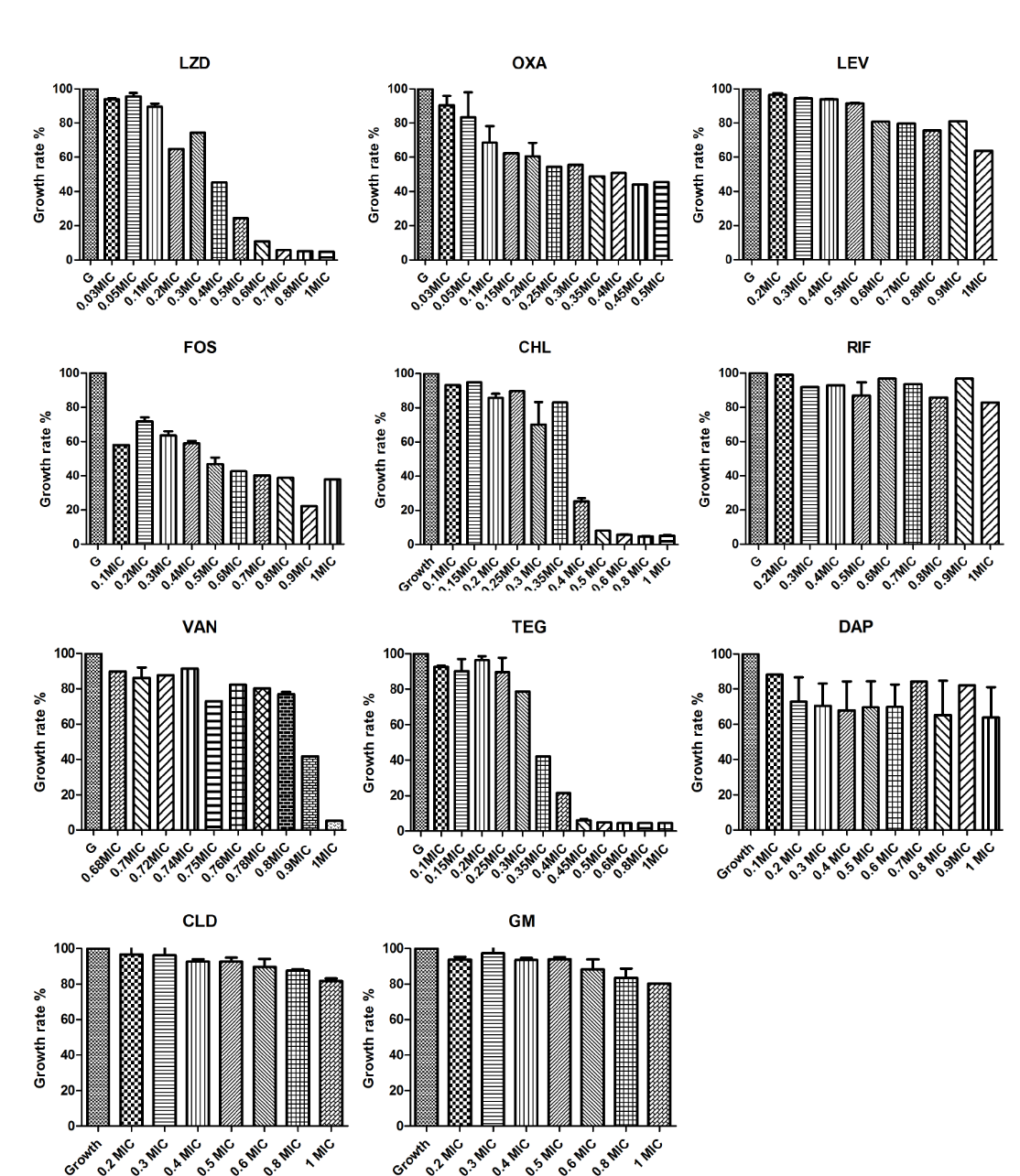


**Figure S1.** Bacterial growth rates versus different concentrations of 11 antibiotics. Drug concentrations are calculated as fold-MIC. LZD, linezolid; OXA, oxacillin; LEV, levofloxacin; FOS, fosfomycin; CHL, chloramphenicol; RIF, rifampicin; VAN, vancomycin; TEG, tigecycline; DAP, daptomycin; CLD, clindamycin; GM, gentamycin.


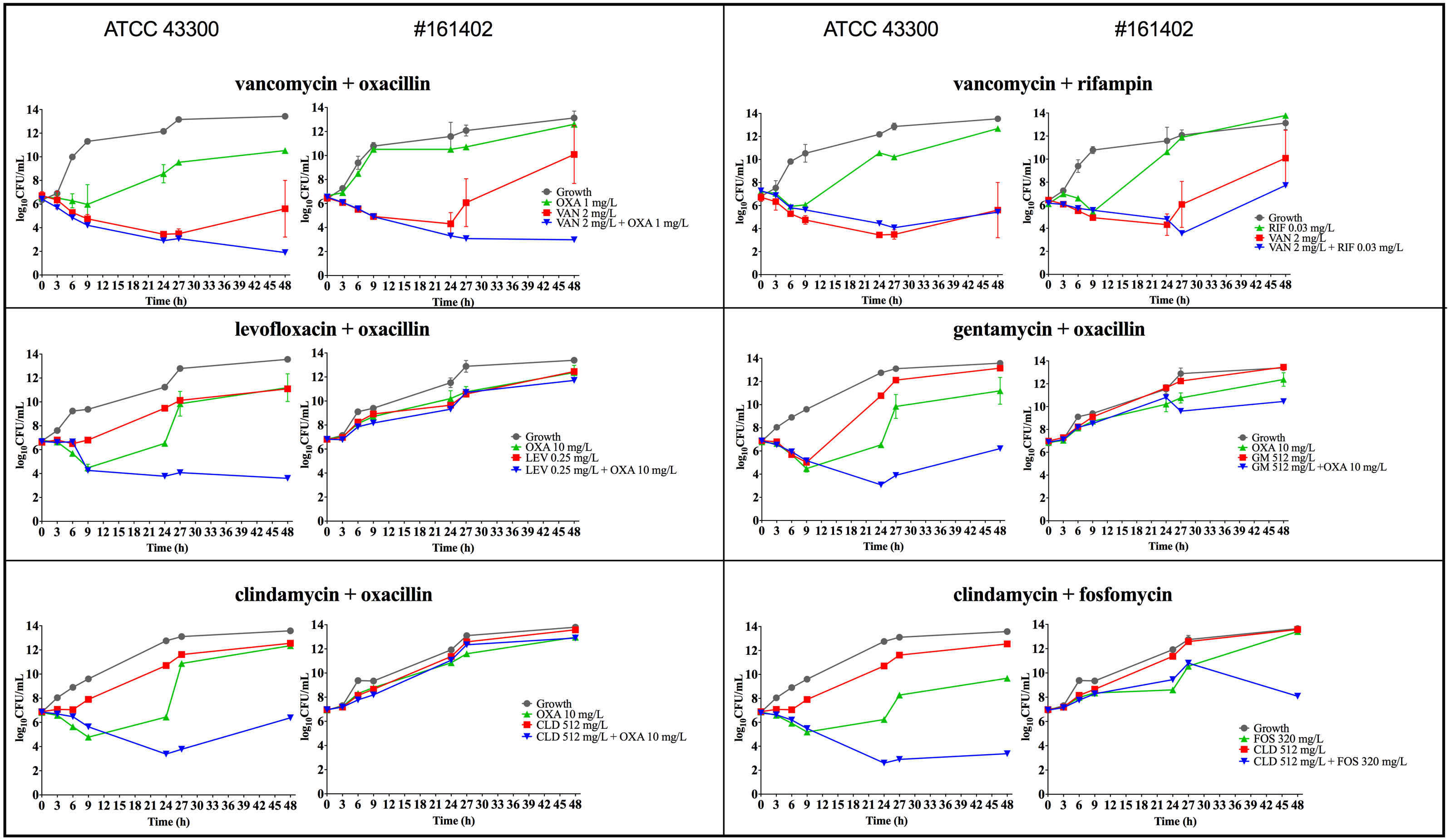


**Figure S2.** In vitro dynamic killing activity of 6 pairwise combinations against both ATCC 43300 and #161402 isolate. Each panel represents the relative combined antibiotics. Bacteria used are labelled at the top of each column.
